# Association Between Spending and Survival of Chronic Heart Failure Across Veterans Affairs Medical Centers

**DOI:** 10.1001/jamanetworkopen.2019.7238

**Published:** 2019-07-19

**Authors:** Peter W. Groeneveld, Elina L. Medvedeva, Lorrie Walker, Andrea G. Segal, Diane M. Menno, Andrew J. Epstein

**Affiliations:** 1 Department of Veterans Affairs Center for Health Equity Research and Promotion, Corporal Michael J. Crescenz Veterans Affairs Medical Center, Philadelphia, Pennsylvania; 2 Division of General Internal Medicine, Department of Medicine, University of Pennsylvania School of Medicine, Philadelphia; 3 Cardiovascular Outcomes, Quality, and Evaluative Research Center, University of Pennsylvania, Philadelphia; 4 Leonard Davis Institute of Health Economics, University of Pennsylvania, Philadelphia; 5 Jazz Pharmaceuticals, Inc, Philadelphia, Pennsylvania; 6 Medicus Economics, LLC, Milton, Massachusetts

## Abstract

**Importance:**

The US Department of Veterans Affairs (VA) provides health care to more than 2 000 000 veterans with chronic cardiovascular disease, yet little is known about how expenditures vary across VA Medical Centers (VAMCs), or whether VAMCs with greater health expenditures are associated with better health outcomes.

**Objectives:**

To compare expenditures for patients with chronic heart failure (CHF) across the nation’s VAMCs and examine the association between health care spending and survival.

**Design, Setting, and Participants:**

Retrospective cohort study using existing administrative data sets from the VA’s Corporate Data Warehouse and each veteran’s Medicare enrollment information and claims history for fee-for-service clinicians outside of the VA from 265 714 patients diagnosed with CHF between April 1, 2010, and December 31, 2013, who received care at any of 138 VAMCs or affiliated outpatient clinics nationwide. Patients were followed up through September 30, 2014. Data were analyzed from April 1, 2010, through September 30, 2014.

**Main Outcomes and Measures:**

Main outcomes were patient deaths per calendar quarter and aggregate VA costs per calendar quarter. Hierarchical generalized linear models with hospital-level random effects were estimated to calculate both risk-standardized annual health care expenditures and risk-standardized annual survival rates for veterans with CHF at each VAMC. The association between VAMC-level expenditures and survival was then modeled using local and linear regression.

**Results:**

Of the 265 714 patients included, 261 132 (98.7%) were male; 224 353 (84.4%) were white; 41 110 (15.5%) were black, Asian, Pacific Islander, American Indian, or Alaskan Native; and 251 (0.1%) did not report race. Mean (SD) age of the patients included was 74 (10) years. Across 138 VAMCs, mean (95% CI) annual expenditures for veterans with CHF varied from $21 300 ($20 300-$22 400) to $52 800 ($49 400-$54 300) per patient, whereas annual survival varied between 81.4% to 88.9%. There was a modest *V*-shaped association between spending and survival such that adjusted survival was 1.7 percentage points higher at the minimum level of spending compared with the inflection point of $34 100 per year (*P* = .001) and 1.9 percentage points higher at the maximum level of spending compared with the inflection point (*P* = .006).

**Conclusions and Relevance:**

Despite marked differences in mean annual expenditures per veteran, only a modest association was found between CHF spending and survival at the VAMC level, with slightly higher survival observed at the extremes of the spending range. Hospitals with high expenditures may be less efficient than their peer institutions in producing optimal health outcomes.

## Key Points

**Question:**

What is the association between health care spending and survival in patients with chronic heart failure across US Veterans Affairs Medical Centers?

**Findings:**

This cohort study of 265 714 patients found that mean annual expenditures varied from $21 300 to $52 800 per patient, and annual survival varied between 81.4% and 88.9%. There was a modest, statistically significant *V*-shaped association between spending and survival; however, the general association between spending and survival was weak.

**Meaning:**

Several Veterans Affairs Medical Centers with high expenditures may be less economically efficient than their peer institutions in producing good health outcomes in their patients with chronic heart failure.

## Teaser

This cohort study uses data from more than 265 000 US veterans treated at Veterans Affairs Medical Centers (VAMCs) to compare health care spending for patients with chronic heart failure across VAMCs and to examine the association between spending and survival.

## Introduction

Chronic heart failure (CHF) is prevalent among 5% of US veterans in the Department of Veterans Affairs (VA) health care system,^1^ and annual mortality among those with CHF is 15%.^2^ The health care costs for patients with CHF are both high^3^ and highly variable in non-VA patient populations.^4^ High CHF expenditures are associated with both the high frequency of hospitalizations^4^ and the costs of testing, pharmacotherapy, devices, and chronic disease management programs.^5^ Small area variation in health care spending for the population of patients with CHF and others has been well documented,^6,7,8^ including variation in spending across the VA.^9^ Although comparisons of VA costs and outcomes in highly selected VA clinical populations have been previously reported,^10^ relatively little is known about whether and how the variation in health care spending among large patient populations with chronic disease across the VA is associated with the substantial variation in cardiovascular outcomes in these populations.^1,11^ In particular, it is unknown whether VA Medical Centers (VAMCs) with greater than average spending per veteran achieve better than average survival rates.

In contrast to privately managed health systems predominantly operating under fee-for-service reimbursement, the VA has a fixed, global budget established by an annual Congressional appropriation, and each VAMC nationwide ultimately receives an annual funding allocation. Although the VA’s national health care system is highly integrated and many costs (eg, pharmaceutical prices and wage scales) are uniformly established at the national level,^12^ there remains ample flexibility in how each VAMC allocates resources across patient care services. Because VA clinicians inherently cannot increase the VA’s revenues either by delivering greater volumes of care or preferentially delivering more profitable health care services, it is essential that the VA’s limited health care dollars be used efficiently.

The goal of our study was to examine variation in CHF spending across the VA from April 1, 2010, through September 30, 2014, and to assess whether VAMCs with higher per-patient spending produced better CHF survival rates.

## Methods

This study followed the Strengthening the Reporting of Observational Studies in Epidemiology (STROBE) reporting guideline.^13^ In the first part of this study, we estimated VAMC-level risk-standardized annual spending and risk-standardized survival per CHF patient, via separate multivariable models of costs and survival among veterans with CHF during consecutive discrete (ie, 3-month) intervals from April 1, 2010, through September 30, 2014. In the study’s second part, we analyzed the association between these 2 VAMC-level estimates. The institutional review board of the Corporal Michael J. Crescenz VAMC approved the study protocol and also waived informed consent requirements owing to use of deidentified data.

### Data Sources

Administrative health care data from October 1, 2009, through September 30, 2014, were obtained from the VA’s Corporate Data Warehouse, a national data repository of inpatient, outpatient, laboratory, and pharmacy encounters throughout the VA health care system, as well as fee-basis care paid for but provided outside of the VA. We also obtained each veteran’s Medicare enrollment information, as well as inpatient facility, outpatient facility, and clinician fee-for-service claims submitted to Medicare during the same 5-year window. Data on each VAMC’s patient volume and technological capacity were obtained from VA internal operational reports.

### Cohort Selection

We identified veterans as having CHF if they had at least 1 VA health care encounter with *International Classification of Diseases, Ninth Revision, Clinical Modification* codes 398.91, 402.01, 402.11, 402.91, 404.01, 404.03, 404.11, 404.13, 404.91, 404.93, 425.4, 428.0, 428.1, 428.20-428.23, 428.30-42833, 428.40-428.43, or 428.9. Patients whose earliest qualifying diagnosis occurred between October 1, 2009, and March 31, 2010, and who were alive on April 1, 2010, entered the cohort in the first calendar quarter of our 4.5-year outcomes observation window (ie, April 1, 2010, to September 30, 2014), ensuring that all cohort patients had at least 6 months’ of previous health care data for accurate coding of comorbidities and recent health events. Patients whose earliest qualifying diagnosis occurred on or after April 1, 2010, but before December 31, 2013, entered the cohort during the calendar quarter of that index diagnosis, and cohort entry ceased after December 31, 2013. Our cohort purposefully included both patients with new onset of heart failure during the study’s observation window and patients with existing CHF to more fully capture the entirety of VAMC-level spending on their patients with CHF. Data were analyzed from April 1, 2010, through September 30, 2014.

### Exclusions

Many VA-enrolled veterans obtain extensive health care from non-VA clinicians via enrollment in Medicare or other health insurance programs. To ensure that we accounted for health care use and costs occurring outside of the VA, for each calendar quarter from April 1, 2010, to September 30, 2014, we restricted the cohort to veterans who were, because of age (>65 years), a qualifying disability, or both, enrolled in fee-for-service Medicare for at least 2 months during the calendar quarter, according to the veteran’s Medicare enrollment data. Veterans who died during the first 2 months of a calendar quarter—and who therefore may have had fewer than 2 months of Medicare coverage—were retained in the cohort if they had Medicare fee-for-service coverage while living.

### Health Care Expenditures

Veterans Affairs health care expenditures were estimated using the VA’s Managerial Cost Accounting database, which assigns a cost to every VA health care encounter by proportionally allocating a VAMC’s actual labor, equipment, supply, overhead, and other operational costs to each inpatient and outpatient encounter.^14^ The Managerial Cost Accounting algorithm is designed so that the sum of each VAMC’s inpatient and outpatient encounter costs for each fiscal year equals that VAMC’s actual clinical operations annual expenditure. Because accounting practices can occasionally generate irrational individual encounter costs (eg, a negative cost, or exorbitant cost that is greatly in excess of possible resource expenditure), for a fewer than 1% of encounters we used an alternative well-validated cost-accounting method developed by the VA’s Health Economics Resource Center, by which a standard Medicare payment amount is attributed to the VA encounter.^15^ All VA health care costs for each veteran in each calendar quarter were extracted, with costs from hospitalizations that spanned more than 1 quarter apportioned relative to the number of hospital days occurring in each quarter. Because CHF likely contributes to multiple reasons for health care encounters (eg, infections, psychiatric illness) that are not labeled as CHF costs in administrative data, we did not attempt to disaggregate costs into heart failure related vs non–heart failure related.

### Survival

The VA’s Death Master File was used to ascertain deaths occurring from April 1, 2010, through September 30, 2014. The Death Master File comprises death data from the VA’s Beneficiary Identification and Records Locator Subsystem database as well as the Social Security Administration Death Master File; it is therefore a highly accurate record of VA-enrolled veterans’ deaths.^16^

### Demographics, Comorbidities, and Previous Events

Each veteran’s age and sex were obtained from the VA’s enrollment database, and self-reported race data were obtained from Medicare’s enrollment files. Comorbidities were assessed using all diagnosis *ICD-9-CM* codes from each veteran’s VA and Medicare administrative claims using the Elixhauser comorbidity^17^ classification scheme, as well as additional chronic conditions derived from hierarchical condition categories typically incorporated in hospital comparison models of cardiovascular disease care^18,19,20^ (ie, cerebrovascular disease, dementia, dialysis, and ischemic heart disease). Each veteran’s VA and Medicare claims also were assessed for major medical or surgical events, including acute myocardial infarction, acute coronary syndrome, percutaneous coronary intervention, coronary artery bypass graft, pneumonia, and stroke.

### Data Organization and Analytic Design for Data Analyses

Data from each of the 18 calendar quarters from April 1, 2010, through September 30, 2014, were organized at the patient-quarter level, and these data were first analyzed with a focus on the death outcome, and then separately analyzed for costs of care. Specifically, the first analysis was a patient-quarter–level discrete-time survival analysis, with the occurrence of death during a calendar quarter as the primary outcome variable. The second analysis was a patient-quarter–level discrete-time cost analysis, with the aggregate costs of VA care during the calendar quarter as the primary outcome variable.

### Comorbidity Variable Coding

Each patient’s vector of binary comorbidity variables was updated in each quarter based on all VA and Medicare health care encounters occurring during that quarter. We assumed that each patient’s chronic comorbid conditions (eg, hypertension, peripheral vascular disease) were present perpetually after their onset, unless the condition and another condition were mutually exclusive (eg, tumor without metastasis and metastatic cancer). The occurrence of selected major medical or surgical events in the previous 2 calendar quarters was assessed for each patient and updated quarterly.

### VA Hospital Assignment and Censoring

We attributed to each VAMC all the health care provided by either the main VA hospital or its affiliated outpatient clinics. Within each calendar quarter, veterans were assigned to a primary VAMC, defined as the VAMC where the veteran had received the plurality of his/her VA health care, quantified as inpatient hospital days plus outpatient clinic visits. In cases where a veteran had more than 1 VAMC meeting this criterion, that veteran was assigned to the VAMC that provided care most proximal in time to the close of the calendar quarter. If a veteran had no VA encounters during a quarter, he or she was assigned to his or her primary VAMC during the previous calendar quarter. Veterans were censored from the study cohort after 2 consecutive calendar quarters with no VA encounters. Censored patients who subsequently reinitiated VA care were rejoined to the study cohort during the quarter when their VA health care resumed.

### Statistical Analysis

#### Patient-Level Outcomes and Expenditure Models

For regression model with mortality as the dependent variable, as well as the regression model with aggregate expenditure as the dependent variable, we estimated hierarchical generalized linear models with hospital random effects (assuming normal distributions), analogous to the methods used by the Centers for Medicare & Medicaid Services in their Hospital Compare methodology.^20,21^ These models included annual fixed effects to control for temporal changes in outcomes independent of the location of care, and an indication that the individual was in the cohort at inception (the first quarter necessarily included patients who had long-standing CHF, whereas patients joining the cohort in subsequent quarters were more likely to have incipient disease). Models used a complementary log-log link function as is typical for interval-censored survival models of this type.^22^

The hospital-level random effects estimated by the hierarchical model were used to calculate a risk-standardized mortality ratio for each hospital, using the Hospital Compare formula (ie, the hospital’s predicted mortality as calculated from the observed outcome rate, with Bayesian shrinkage applied to the estimate to account for instability of rate estimators in small samples, divided by the expected mortality as derived from the observed characteristics of each hospital’s patients).^21^ Confidence intervals for each hospital’s risk-standardized mortality estimate were derived using 380 bootstrap replications of the patient-level data set to ensure that each hospital was included in at least 200 replications, with random selection at the hospital level, following the methods for confidence interval derivation described by Ash et al.^21^

#### VAMC-Level Analyses

The cohort consisted of the VAMCs with risk-standardized annual spending and mortality estimates calculated in the study’s first part. These were supplemented with variables representing organizational environment and capabilities, including each VAMC’s complexity (ie, VA’s multitier hospital classification system based on the diversity of clinical services, teaching status, and referral behavior, which was simplified to 3 levels: standard, intermediate, and complex) and US Census region (Northeast, South, Midwest, West). We also calculated the mean (across quarters) fraction of total health care received by the VAMC’s CHF cohort that was provided by the VA, by dividing the cohort’s quarterly number of VA outpatient encounters and hospital days by the total (VA- plus Medicare-reimbursed) quarterly outpatient encounters plus hospital days.

In preparation for modeling, mortality was inverted to reflect risk-standardized annual survival (ie, 100% minus mortality rate), and risk-standardized annual spending was adjusted for variation across VAMCs in the price of labor. Labor price adjustments were made by dividing 50% of each VAMC’s spending by the multiplier used by the federal government to locally adjust salary rates at federal facilities.^23^ The other 50% of each VAMC’s spending was attributable to capital expenses, which were assumed not to vary across VAMCs (eg, as a result of national purchasing contracts). To check the robustness of this assumption, analyses were repeated after attributing 0%, and then 100%, of spending to inputs that would vary in price with the local price of labor.

To explore the spending-survival association nonparametrically, we first estimated a Spearman ρ correlation coefficient and locally weighted scatterplot smoothing regression. After locally weighted scatterplot smoothing estimation, we estimated an adjusted linear regression model that controlled for VAMC complexity, geographic region, and the share of each VAMC’s health care for veterans with CHF that was delivered outside the VA. We then assessed whether a purely linear association vs linear spline for cost was the best fit to the data via a likelihood ratio test. Cost was ultimately represented as a linear spline with a single knot, which was identified via an auxiliary joinpoint regression using methods described by Bacon and Watts.^24^ The models were weighted by the number of unique veterans in each VAMC’s CHF cohort to address heteroskedasticity from variation in the precision of the first-stage estimates of spending and survival; SEs were also made robust to heteroskedasticity of unknown form.

All analyses were conducted using SAS version 9.4 (SAS Institute Inc) and Stata version 15.1 (StataCorp LLC) statistical software. All statistical tests were 2-sided, and we assumed *P* < .05 indicated statistical significance.

## Results

Of the 265 714 patients studied, 261 132 (98.7%) were male; 224 353 (84.4%) were white; 41 110 (15.5%) were black, Asian, Pacific Islander, American Indian, or Alaskan Native; and 251 (0.1%) did not report race. We excluded data from 6 VAMCs with fewer than 200 qualifying patients, because low patient volume made accurate mortality and expenditure estimation at these hospitals infeasible. After exclusions, we identified 265 714 veterans with CHF who were dually enrolled in the VA and fee-for-service Medicare (Table 1), among a total of 348 015 veterans with CHF, hence our cohort comprised 76.4% of all veterans with CHF. These veterans received care at 138 distinct VAMCs, located in all 50 states and the District of Columbia.

**Table 1.** Characteristics of the Department of Veterans Affairs Cohort of 265 714 Patients With Chronic Heart Failure^a^

Characteristic	No. (%)^b^
Age, mean (SD), y	74 (10)
Male	261 132 (98.3)
Race^c^	
White	224 353 (84.4)
Black, Asian, Pacific Islander, American Indian, or Alaskan Native	41 110 (15.5)
Unknown	251 (0.1)
Date entering cohort^d^	
April 1, 2010, to September 30, 2010	154 368 (58.1)
October 1, 2010, to September 30, 2011	43 928 (17.5)
October 1, 2011, to September 30, 2012	32 799 (12.3)
October 1, 2012, to September 30, 2013	28 248 (10.6)
October 1, 2013, to December 31, 2013^e^	6371 (2.4)
Clinical comorbidities	
Hypertension	230 073 (86.6)
Coronary artery disease without recent myocardial infarction	144 705 (54.5)
Diabetes without chronic complications	118 012 (44.4)
Chronic pulmonary disease	102 542 (38.6)
Deficiency anemias	77 054 (29.0)
Chronic kidney disease	76 927 (29.0)
Fluid and electrolyte disorders	60 032 (22.6)
Obesity	57 083 (21.5)
Peripheral vascular disease	56 318 (21.2)
Diabetes with chronic complications	54 501 (20.5)
Depression	48 686 (18.3)
Valvular heart disease	37 287 (14.0)
Solid tumor without metastasis	35 433 (13.3)
Hypothyroidism	32 809 (12.3)
Other neurological disorders	29 504 (11.1)
Dementia	24 211 (9.1)
Cerebrovascular disease without recent stroke	20 006 (7.5)
Pulmonary circulation disorders	19 720 (7.4)
Coagulation disorders	17 976 (6.8)
Alcohol abuse	16 557 (6.2)
Weight loss	14 865 (5.6)
Chronic liver disease	9898 (3.7)
Drug abuse	8181 (3.1)
Paralysis	7507 (2.8)
Rheumatoid arthritis or collagen vascular disease	6946 (2.6)
Dialysis	5497 (2.1)
Metastatic cancer	4194 (1.6)
Lymphoma	3783 (1.4)
Blood loss anemia	3108 (1.2)
HIV/AIDS	866 (0.3)
Peptic ulcer disease with bleeding	192 (0.1)
Medical or surgical events during the 6 mo prior to cohort entry	
Stroke	13 227 (5.0)
Acute myocardial infarction or acute coronary syndrome	11 380 (4.3)
Pneumonia	3103 (1.2)
Percutaneous coronary intervention	2926 (1.1)
Coronary artery bypass grafting	1524 (0.6)

^a^ Clinical characteristics on the date of cohort entry.

^b^ Percentages may not sum to 100% owing to rounding.

^c^ Veterans’ race was determined by self-report from their Medicare or Social Security Enrollment data.

^d^ Federal fiscal years begin annually on October 1.

^e^ Enrollment numbers are lower in the final fiscal year because enrollment ceased after the first 3 months.

### CHF Cohort’s Risk-Standardized Expenditure and Survival Variation Across VAMCs

The mean annual VA expenditure per veteran with CHF was $31 400. Risk-standardized annual expenditures varied across the 138 VA hospitals from $21 300 (95% CI, $20 300-$22 400) to $52 800 (95% CI, $49 400-$54 300). Risk-standardized CHF survival varied across the 138 VA hospitals from 81.4% (95% CI, 79.7%-82.9%) to 88.9% (95% CI, 87.6%-89.9%).

### VAMC-Level Association Between Expenditures and Survival

Thirteen VAMCs in the lowest quartile for cost were simultaneously in the highest quartile for survival, whereas 10 VAMCs in the highest quartile for cost were simultaneously in the lowest quartile for survival. Overall, there was a modest negative correlation between risk standardized costs and survival (Spearman ρ = −0.24; *P* = .004).

### VAMC-Level Adjusted Survival-Expenditure Association

After adjusting 50% of annual spending for variation in the local wage index, annual VAMC-level spending ranged from $21 286 to $48 870. Locally weighted scatterplot smoothing regression suggested a slight *V* shape to the spending-survival association, with locally weighted scatterplot smoothing–based spending at a minimum when annual spending was $34 100 (Figure). A likelihood ratio test confirmed the spline model as a better fit (likelihood ratio, 44.75; *P* < .001). Across the span of spending from its minimum ($21 286) to the spline knot where survival was minimized ($34 100), mean adjusted survival at VAMCs was 0.13 percentage points lower (95% CI, 0.06 to 0.21) for each additional $1000 of spending (Table 2).^25^ From the spline knot to its maximum ($48 870), mean adjusted survival at VAMCs was 0.14 percentage points higher (95% CI, 0.05 to 0.23) for each additional $1000 of additional spending. Adjusted survival was 1.7 percentage points higher at the minimum level of spending compared with the spline knot at $34 100 per year (*P* = .001), and 1.9 percentage points higher at the maximum level of spending compared with the spline knot (*P* = .006). Results from the model robustness checks in which the share of annual spending attributable to labor expenses was varied between 0% and 100% were substantively unchanged (Table 2).

**Figure.**
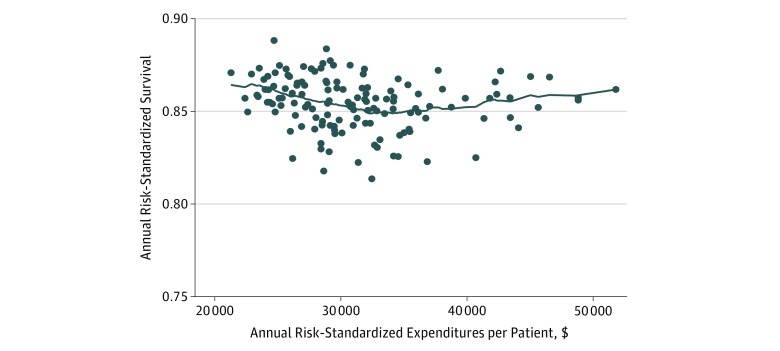
Risk-Standardized Expenditure vs Risk-Standardized Chronic Heart Failure Survival The line indicates the locally weighted scatterplot smoothing estimate of the association between risk-adjusted costs and risk-adjusted survival. Risk-standardized annual survival is calculated as 100% minus mortality rate.

**Table 2.** Hospital-Level Multivariable Model Results

Characteristic	Base Model		Model With 0% Locality Cost Adjustment		Model With 100% Locality Cost Adjustment	
	Coefficient (95% CI)^a^	*P* Value	Coefficient (95% CI)	*P* Value	Coefficient (95% CI)	*P* Value
CHF cost, $ thousands^b^						
Spline 1, minimum cost to knot	−0.13 (−0.06 to −.21)	.001	−0.12 (−0.04 to −0.19)	.003	−0.16 (−0.08 to −0.25)	<.001
Spline 2, knot to maximum cost	0.14 (0.05 to 0.23)	.006	0.11 (0.04 to 0.19)	.007	0.16 (0.05 to 0.26)	.01
Percentage of care outside VA^c^	0.06 (−0.001 to 0.08)	.06	0.04 (0.001 to 0.08)	.04	0.03 (−0.009 to 0.07)	.08
VAMC complexity^d^						
Medium	−0.51 (−1.02 to −0.001)	.049	−0.51 (−1.03 to 0.001)	.051	−0.48 (−0.98 to 0.03)	.07
Low	−0.67 (−1.38 to 0.04)	.06	−0.66 (−1.37 to 0.06)	.07	−0.68 (−1.39 to 0.02)	.06
US Census region^e^						
Midwest	−0.52 (−1.19 to 0.15)	.13	−0.51 (−1.20 to 0.17)	.14	−0.55 (−1.20 to 0.11)	.10
South	−1.34 (−1.86 to −0.82)	<.001	−1.35 (−1.88 to −0.82)	<.001	−1.35 (−1.86 to −0.84)	<.001
West	−0.90 (−1.72 to −0.08)	.03	−0.92 (−1.77 to −0.08)	.03	−0.88 (−1.71 to −0.06)	.04

Abbreviations: CHF, chronic heart failure; VA, Department of Veterans Affairs; VAMC, Veterans Affairs Medical Center.

^a^ Coefficients indicate the percentage point change in survival per unit increase in the characteristic.

^b^ Model was fitted as a piecewise linear regression with the location of the “knot” (spline breakpoint) determined empirically to minimize total model residuals.^23^ The x-location of the knot was $34 100 in the base case model, $34 290 in the 0% locality cost-adjusted model, and $33 122 in the 100% locality cost-adjusted model.

^c^ Defined as the VAMC-level volume of care (hospital days plus outpatient encounters) received by veterans in the CHF cohort outside the VA as a proportion of the total volume of care received at both VA and non-VA facilities.

^d^ Institutional complexity using VA’s rubric assigning hospitals as high (complexity level 1A), medium (complexity level 1B or 1C), or low (complexity level 2 or 3).^25^ Coefficients are compared with high-complexity VAMCs.

^e^ Coefficients are compared with the Northeast region of the US Census.

## Discussion

We observed marked variation in risk-standardized health care expenditures across 138 VA medical centers among veterans with CHF, with annual expenditures at the highest-cost VAMC being approximately 2.5 times greater than expenditures at the lowest-cost VAMC. We also found that risk-standardized per-patient spending had a very modest *V*-shaped association with survival rates among veterans with CHF, such that survival was greater (by approximately 2 percentage points) when spending was near its minimum and its maximum; however, the overall association between spending and outcomes suggested only weak correlation. Addition of hospital-level factors to the regression confirmed that the association between VA spending and outcomes was minimally associated with differences in a VAMC’s local labor costs, structural capacity, or the fraction of health care provided by non-VA sources to each VAMC’s population of patients with CHF.

The tenuous association between health care spending and outcomes was similar to findings of several earlier studies. Although there is extensive variation in health care spending across the United States,^26^ Tsugawa et al^27^ determined that physicians with greater spending on hospitalized Medicare beneficiaries were not associated with better 30-day survival rates among their patients. Earlier studies have found no clear association between survival after inpatient hospitalization and hospital spending.^28,29,30^ In the VA itself, Ho and colleagues^10^ determined that there was minimal correlation between spending and outcomes among percutaneous coronary intervention recipients at the 60 VAMCs that perform the procedure. The current study extends these findings to the outcomes and costs of larger populations of veterans receiving chronic disease care across extended periods by an integrated health system delivering both inpatient and outpatient services. In congruence with the findings of Ho et al,^10^ we observed little evidence that high-spending VAMCs are delivering better health outcomes to their veterans.

Clearly, the association between health system spending and all-cause mortality is complex, and a sizeable portion of mortality risk in populations is unlikely to be greatly affected by health care but is rather the result of socioeconomic, behavioral, and environmental factors beyond the control of any health care system. We would nevertheless suggest that spending differences of the magnitude we observed appear difficult to justify based on clinical differences in patient populations, geographic differences in labor costs, or socioeconomic differences across metropolitan areas. In particular, our results suggest that greater scrutiny should be applied to hospitals at the high end of the spending distribution, because greater spending is not clearly producing better patient outcomes.

We noted a modest, but statistically significant *V*-shaped association between costs and survival that was not explained by geographic or structural differences across VAMCs. There are many possible explanations for this finding, including the possibility that patients at low-spending VAMCs have unmeasured better health or unobserved sources of health support, which would imply there is actually a positive association between spending and outcomes that would have been more clearly apparent in our data, had it been possible for us to measure additional contributing factors at low-cost VAMCs. An alternative explanation for the improved survival at high-cost VAMCs would be that the VAMCs with the highest spending were located in wealthier US cities with both higher operational costs and more unobservable public health and social support. It is also possible that both of these explanatory phenomena occurred simultaneously.

However, even if a modest causal association between spending and outcomes exists, the dominant message of our findings is the high likelihood that health system inefficiency (ie, unnecessary tests, treatments, and other services) remains a substantial, systemwide problem. This is a particularly worrisome finding in the VA health system, in which the fixed operating budget requires explicit spending trade-offs (eg, excess spending for chronic disease A requires lower spending for chronic disease B). To the extent that inefficient spending in the VA could be redeployed to improve veterans’ health outcomes, particularly in a high-cost/high-mortality condition such as CHF, there may be substantial opportunity to deliver better outcomes to veterans at the same operating cost.

### Limitations

There are several limitations to this study. Comorbidity and disease severity are imperfectly measured using administrative data. Therefore, it is possible that the variations in survival and expenditures we observed were a result of unobserved differences across VAMCs in comorbidity and/or disease severity. Also, variation between hospitals in CHF coding practices may have influenced the size of each hospital’s patient cohort: if patients with mild CHF were more frequently coded as having CHF at some VAMCs, those hospitals’ survival rates may have been higher and their average costs lower than hospitals that were less “aggressive” about CHF coding. However, because there are minimal financial incentives in the VA to upcode or overcode patients, and the VA has a national electronic medical record and a standardized system for administrative coding, we believe coding variation across VAMCs was unlikely to explain our results.

Chronic heart failure is likely to both directly and indirectly affect health care costs, but indirect effects are impossible to measure using administrative data. Because it was infeasible to ascertain whether seemingly unrelated health care encounters (eg, psychiatry, dermatology) were in fact associated with CHF, we made no attempt to disaggregate expenditures related to CHF or cardiovascular care and other health care expenditures. As a result, the high health care spending we observed at some VAMCs may have been unrelated to CHF health care, and thus not reflective of inefficiency in CHF care delivery. Finally, we excluded veterans who were uninsured, had private health insurance, or were enrolled in Medicare Advantage. Although our cohort still comprised 76.4% of all veterans with CHF, our results may have differed if exclusions had not been necessary.

## Conclusions

There was substantial variation in risk-standardized expenditures for CHF across the nation’s VAMCs, with a modest, *V*-shaped association between spending and survival such that mean survival was slightly higher at the lowest and highest levels of spending, but in general the correlation between spending and outcomes was weak. Several VAMCs with high expenditures may be less economically efficient than peer institutions in producing good health outcomes among patients with CHF.
